# Association of Alternative Tobacco Product Initiation With Ownership of Tobacco Promotional Materials Among Adolescents and Young Adults

**DOI:** 10.1001/jamanetworkopen.2019.4006

**Published:** 2019-05-17

**Authors:** Hoda S. Abdel Magid, Patrick T. Bradshaw, Pamela M. Ling, Bonnie Halpern-Felsher

**Affiliations:** 1 Division of Epidemiology, Department of Health Research and Policy, Stanford University, Palo Alto, California; 2 Division of Epidemiology, School of Public Health, University of California, Berkeley; 3 Center for Tobacco Control Research and Education, University of California, San Francisco; 4 Division of Adolescent Medicine, Department of Pediatrics, Stanford University, Palo Alto, California

## Abstract

**Importance:**

Use of alternative tobacco products (ATPs) such as electronic cigarettes, chewing tobacco, pipes, cigars, cigarillos, little cigars, and hookah is rapidly increasing. Although marketing restrictions exist for cigarettes, marketing of ATPs is not yet fully regulated, and studies have not assessed the association between ownership of ATP promotional materials and subsequent ATP or cigarette initiation among adolescents and young adults.

**Objective:**

To estimate the association between marketing receptivity measured at baseline and ATP and any tobacco initiation 1 year later, including cigarettes, among adolescents and young adults.

**Design, Setting, and Participants:**

Longitudinal cohort study of adolescents and young adults aged 13 to 19 years recruited at high schools in California from July 2014 to October 2015, with follow-up 1 year later. Data were analyzed from January to March 2018.

**Exposures:**

Ownership of ATP-specific promotional material and ownership of any tobacco promotional materials (eg, samples, coupons, branded caps, t-shirts, or posters) assessed in wave 1.

**Main Outcomes and Measures:**

Outcomes were (1) ATP initiation and (2) either ATP or cigarette initiation in wave 2.

**Results:**

Of 757 participants (mean [SD] age at wave 1, 16.1 [1.1] years; 481 [63.5%] female; 166 [21.9%] Asian or Pacific Islander, 202 [26.7%] white, and 276 [36.4%] Latino), 129 (17.0%) initiated ATP use and 141 (18.6%) initiated ATP or cigarette use 1 year later. In unadjusted models, wave 2 ATP initiation was found to be significantly associated with wave 1 ownership of ATP promotional materials (odds ratio, 2.23; 95% CI, 1.26-3.97). After adjustment for wave 1 demographic covariates, the association between ownership of ATP promotional material and ATP initiation 1 year later yielded similar results (odds ratio, 2.13; 95% CI, 1.16-3.91). Results of models assessing a combined outcome variable of either ATP or cigarette ever use were not statistically significant.

**Conclusions and Relevance:**

Ownership of ATP promotional materials was associated with subsequent initiation of ATPs. The results of this study are consistent with the suggestion that current marketing restrictions for cigarettes, including restrictions of the distribution of samples, coupons, and other promotional material, should extend to ATPs.

## Key Points

**Question:**

Is ownership of alternative tobacco product (ATP) marketing materials (eg, samples, coupons, branded caps, t-shirts, or posters) associated with subsequent initiation of ATPs or cigarettes among adolescents and young adults?

**Findings:**

In this longitudinal cohort study of 757 adolescents and young adults in California, ownership of ATP promotional materials was associated with ATP initiation.

**Meaning:**

Similar to cigarette marketing, youth who report ownership of ATP promotional material may be more likely to use ATPs.

## Teaser

This cohort study estimates the association between ownership of alternative tobacco product (ATP) promotional materials and initiation of ATP or cigarette use among adolescents and young adults in California.

## Introduction

Although rates of cigarette and other combustible tobacco use have declined among adolescents and young adults in the United States, use of alternative tobacco products (ATPs) such as electronic cigarettes (e-cigarettes or vapes), smokeless tobacco (chewing tobacco, dissolvable or dipping tobacco, moist snuff, and snus), tobacco pipes, cigars (traditional, filtered, and cigarillos or little cigars), and hookah (water pipes) has increased.^1,2,3^ For example, according to the 2018 National Youth Tobacco Survey,^4^ past 30-day e-cigarette use increased among high school students from 1.5% in 2011 to 20.8% in 2018. Among high school participants, e-cigarettes were the most commonly used tobacco product (20.8%), followed by cigars (7.7%), cigarettes (7.6%), smokeless tobacco (5.5%), hookah (3.3%), pipe tobacco (0.8%), and bidis (0.7%).^4^ Among adults, past 30-day use of ATPs was highest among young adults (ages 18-24 years), with e-cigarettes (5.2%), cigars and cigarillos (4.3%), smokeless tobacco (2.9%), and hookah (2.5%) being the most commonly used.^5^ The increase in ATP use among adolescents and young adults in the United States poses a threat to decades of public health campaigns aimed at reducing tobacco use by renormalizing smoking, decreasing risk perceptions, and increasing benefit perceptions of tobacco products.^6,7,8^

This changing landscape of tobacco use may be due in part to the marketing of ATPs online, in newspapers and magazines, in retail stores, on television, in movies, in sports, as part of music event sponsorships, in advertisements placed at children’s eye level, and through ownership of tobacco promotional material such as t-shirts, hats, keychains, posters, and other items with tobacco companies’ insignia and logos.^9,10,11,12,13^ Even though some regulations of ATPs have been implemented, such as the US Food and Drug Administration’s Youth Tobacco Prevention Plan prohibiting e-cigarette sales to minors, ATPs are still widely marketed, including the distribution of ATP samples and coupons via postal mail.^14^ According to a recent analysis of the National Youth Tobacco Survey,^15^ approximately 13% of middle school and high school students reported ownership of e-cigarette coupons in the past 30 days received through digital communications or postal mail.

Tobacco marketing receptivity has been measured by examining individuals’ (1) exposure and affective response to advertisements and/or (2) ownership of tobacco promotional materials.^16,17,18^ Most studies on marketing receptivity among adolescents have focused primarily on the former measurement of marketing receptivity rather than the latter. Four cross-sectional studies have examined the association of marketing receptivity to tobacco products, as measured by receipt of coupons, samples, or promotional items, with cigarette use among adolescents^15,19,20^ and young adults^21^ in the United States, with 1 UK study^22^ focusing on adolescents. These studies showed positive associations between current cigarette smoking, awareness of coupons,^22^ and receipt of coupons among adolescents and young adults.^15,19,20,21^ Receipt of tobacco coupons was also associated with increased positive smoking-related beliefs, higher susceptibility to smoking, lower likelihood of confidence in quitting smoking, and higher likelihood of intentions to purchase tobacco in the future.^19^

We know of no study that has longitudinally examined how ownership of ATP promotional items is associated with subsequent initiation of ATPs among adolescents and young adults. In this longitudinal study, we explored the association between ATP marketing receptivity and initiation of different ATPs, including e-cigarettes; chewing tobacco or moist snuff (smokeless tobacco); tobacco pipes; cigars, cigarillos, or little cigars; and hookah in a California cohort of adolescents and young adults. We assessed marketing receptivity, defined as both ownership of (1) ATP promotional items and (2) ATP or cigarette promotional items. These promotional items included samples, coupons, and branded material (eg, caps, t-shirts, or posters) self-reported at baseline. We then examined the association between marketing receptivity with both (1) ATP initiation and (2) ATP or cigarette initiation 1 year later. We hypothesized that adolescents who owned promotional materials would be more likely to initiate ATP and cigarette use.

Findings of this study may help inform state and federal tobacco marketing regulations. Current regulations such as the 1998 Master Settlement Agreement and the Family Smoking Prevention and Tobacco Control Act implement restrictions on cigarette marketing, including the distribution of cigarette promotional materials to adolescents during public events such as instructional seminars and music and sporting events as well as through postal mail and loyalty programs.^14,23,24,25,26^ Although some of these marketing strategies, including the distribution of free samples, sales in vending machines, and the minimum sales age, are regulated for select ATPs such as e-cigarettes, many marketing restrictions do not currently extend to ATPs.^10,27^

## Methods

### Data and Study Design

The data presented in this analysis are from a survey conducted in 2 waves, wave 1 (July 2014 to October 2015) and wave 2 (July 2015 to March 2016), of a prospective longitudinal study of adolescents in ninth and twelfth grades. Students were recruited from 10 California high schools with diverse student populations. This study was approved by Stanford University’s institutional review board. Assent forms were signed by the student and consent forms were signed by their parent or guardian. Students aged 18 years or older provided their own consent. This study follows the Strengthening the Reporting of Observational Studies in Epidemiology (STROBE) reporting guideline for cohort studies.^28^

Approximately 4000 students received materials about the study, with 1299 (32%) returning signed consent forms. Of these students, 405 (31.1%) were disqualified from the study because they provided invalid contact information, were otherwise ineligible (eg, being in the wrong grade), or could not be contacted by the researchers. Overall, 772 eligible consented participants (86.4%) completed the survey in wave 1 and 578 (64.1%) completed the survey in wave 2. Compared with the schools from which we sampled, our final sample had more female and fewer male participants and a greater percentage of Asian participants. Tobacco use rates and patterns were similar to those in national data sets.^6,29^

Consenting individuals were sent an email with a link to the online surveys, administered through Qualtrics. Data for this analysis (n = 757) represent all individuals with complete data on exposure, covariates (wave 1), and outcome variables (wave 2). Participants received $10 for participating in wave 1 and $15 for wave 2. Study details have been previously published.^8^ Study participants were compared with the overall student bodies at each school, with no significant differences found.^6,8,30,31^

### Measures

All questions listed in this section were asked for the following products: e-cigarettes; cigarettes; smokeless tobacco (chewing tobacco or moist snuff); cigars, cigarillos, or little cigars; and hookah. With the exception of cigarettes, we refer to these products as *ATPs*.

#### Use of ATPs and Cigarettes Ever and in the Past 30 Days

In each wave, participants were asked whether they had ever used cigarettes and ATPs. Participants were asked, “During your entire life, how many times have you ever used [product]?” Two variables were created from this set of questions: (1) ATP ever use and (2) ATP or cigarette ever use. The variable of ATP ever use was dichotomized as yes vs no, indicating whether the participant reported ever use of at least 1 ATP. The variable of ATP or cigarette ever use was dichotomized to indicate whether participants reported any lifetime use of any tobacco product (including cigarettes).

In each wave, participants were asked, “During the last 30 days, on about how many days did you use [product]?” Two variables were created from this set of questions: (1) ATP past-30-day use and (2) ATP or cigarettes past-30-day use. The variable of ATP past-30-day use was dichotomized as yes vs no to indicate whether a participant reported any past use of at least 1 ATP in the past 30 days. The variable of ATP or cigarette past-30-day use was dichotomized as yes vs no to indicate whether participants reported any use of any tobacco product (including cigarettes) in the past 30 days.

#### ATP Initiation

Our primary outcome of interest was ATP initiation between wave 1 and wave 2. Individuals were categorized as having initiated ATP between waves 1 and 2 if they reported (1) no ever or past-30-day ATP use in wave 1 and (2) either ever or past-30-day ATP use on the wave 2 survey. Our secondary outcome of interest was similarly defined for ATP or cigarette initiation.

#### Receipt of Product Coupons and Samples

Participants were asked, “Have you received coupons for any of the products listed below [e-cigarettes, chewing or dipping tobacco or moist snuff, tobacco pipes, cigars, cigarillos or little cigars, and hookahs]?” coded as yes vs no and (2) “Have you received samples for any of the products listed below?” coded as yes vs no.

#### Ownership of Promotional Materials

Participants were asked, “Do you or your friends own any promotional materials (such as caps, t-shirts, posters) for the products listed below [e-cigarettes, chewing or dipping tobacco or moist snuff, tobacco pipes, cigars, cigarillos or little cigars, and hookahs]?” Response options included “No, neither I nor my friends own items,” “Yes, I own items,” and “Yes, my friends own items.” Individuals were categorized as owning promotional material if they responded “Yes, I own items,” with responses coded as yes vs no.

We characterized individual exposure to marketing in 2 ways. First, we defined ownership of ATP-specific promotional materials (yes or no) if students reported at least 1 of the following: receipt of ATP product samples, receipt of ATP product coupons, or ownership of ATP product promotional material for any ATP. Second, we defined ownership of ATP or cigarette promotional materials (yes or no) if students reported at least 1 of the following: receipt of any product samples, receipt of any product coupons, or ownership of any product promotional material from cigarettes or any ATP product. These measures are based on established literature showing ownership of promotional materials as an indicator of tobacco marketing receptivity.^16,17^

#### Demographic Characteristics

At wave 1, participants self-reported age in years, sex, race/ethnicity, and mother’s educational level. Age was dichotomized into ages 13 to 15 and 16 to 19 years to distinguish between younger adolescents, older adolescents, and young adults in our sample. Race/ethnicity was measured in 11 categories and recoded into 4 categories (non-Hispanic white, non-Hispanic Asian/Pacific Islander, Latino, and non-Hispanic other), representing the most prevalent demographic makeup of the schools in California.

### Statistical Analysis

We estimated odds ratios (ORs) of the relationship between ownership of (1) ATP promotional materials and (2) ATP or cigarette promotional materials reported in wave 1 with (1) ATP initiation and (2) ATP or cigarette initiation in wave 2. Generalized estimating equation logistic regression models were used in all analyses to account for potential clustering by school and to estimate population-averaged parameter estimates with robust standard errors with assumed exchangeable working correlation matrix.^32,33,34^ We present parameter estimates (ORs and corresponding 95% confidence intervals) for the association of ownership of ATP promotional materials with (1) ATP initiation and (2) ATP and cigarette initiation.

Individual demographic and socioeconomic characteristics determined a priori to be associated with our exposure and outcome based on prior literature were considered as confounders in the multivariate analysis.^15,16,18,19,20,21,22^ We adjusted for 2 covariate sets to separately assess the influence of demographic, socioeconomic, and behavioral factors in our analysis. Model 1 adjusts for baseline age, sex, race/ethnicity, and mother’s educational level; model 2 adjusts for model 1 covariates plus cigarette use ever and alcohol use ever at baseline. Data analysis was conducted using R statistical software version 3.2.1 (R Project for Statistical Computing).

## Results

In wave 1 (757 participants), adolescents had a mean (SD) age of 16.1 (1.1) years and 481 (63.5%) were female. In all, 166 participants (21.9%) identified as Asian or Pacific Islander, 202 (26.7%) as white, and 276 (36.4%) as Latino. In wave 2, 129 participants (17.0%) subsequently initiated ATP use and 141 (18.6%) initiated either ATP or cigarette use. A full description of the sample by tobacco initiation status is provided in Table 1. Among the 81 participants who reported ownership of any cigarette or ATP promotional item, most reported ownership of promotional items specific to cigarettes, e-cigarettes, or hookah. Table 2 further describes marketing receptivity from cigarettes and ATPs.

**Table 1.** Descriptive Baseline Characteristics of Participants at Wave 2 Overall, by ATP Initiation Status, and by ATP and Cigarette Initiation Status^a^

Covariate	Participants, No. (%)			
	Total Sample (N = 757)	Never Used ATP or Cigarettes (n = 628)	ATP Initiation (n = 129)	ATP or Cigarette Initiation (n = 141)
Age				
13-15 y	304 (40.2)	274 (43.6)	30 (23.2)	32 (22.7)
16-19 y	453 (59.8)	354 (56.3)	99 (76.7)	109 (77.3)
Sex				
Male	276 (36.5)	234 (37.2)	42 (32.5)	45 (31.9)
Female	481 (63.5)	394 (62.7)	87 (67.4)	96 (68.1)
Race/ethnicity				
White	202 (26.7)	174 (27.7)	28 (21.7)	30 (21.2)
Asian or Pacific Islander	166 (21.9)	139 (22.1)	27 (20.9)	25 (17.7)
Latino	276 (36.4)	227 (36.1)	49 (37.9)	57 (40.4)
Other	113 (14.9)	88 (14.01)	25 (19.4)	29 (20.5)
Mother’s education				
Don’t know	73 (9.6)	60 (9.5)	13 (10.1)	13 (9.2)
Elementary or junior high school	68 (8.9)	57 (9.0)	11 (8.5)	18 (12.7)
Some high school	68 (8.9)	54 (8.6)	14 (10.8)	15 (10.6)
High school graduate or GED	131 (17.3)	86 (15.3)	35 (27.1)	38 (26.9)
Some college	127 (16.8)	111 (17.7)	16 (12.4)	22 (15.6)
2-y college degree	75 (9.9)	58 (9.2)	17 (13.1)	17 (12.0)
4-y college degree	123 (16.2)	112 (17.8)	11 (8.5)	10 (7.1)
Graduate or professional degree	92 (12.1)	80 (12.7)	12 (9.3)	8 (5.6)
Ever cigarette use	95 (12.5)	NA	17 (13.1)	19 (13.5)
Ever alcohol use	370 (48.8)	281 (44.7)	89 (68.9)	93 (65.9)
Ownership of ATP promotional item	64 (8.4)	45 (7.1)	19 (14.7)	16 (11.3)
Ownership any cigarette or ATP promotional item	81 (10.7)	58 (9.2)	23 (17.8)	22 (15.6)

Abbreviations: ATP, alternative tobacco product; GED, general education diploma; NA, not applicable.

^a^ Alternative tobacco products including electronic cigarettes (e-cigarettes); chewing or dipping tobacco or moist snuff; tobacco pipes; cigars, cigarillos, or little cigars; and hookah.

**Table 2.** Ownership of Cigarette and Alternative Tobacco Product Promotional Items by Cigarette and Alternative Tobacco Product Use Among 757 Adolescents and Young Adults

Promotional Item	Participants, No.					
	Cigarettes	E-cigarettes	Chewing or Dipping Tobacco or Moist Snuff	Tobacco Pipes	Cigars, Cigarillos, or Little Cigars	Hookah
Samples	6	11	1	0	2	13
Coupons	25	24	5	1	7	12
Other promotional items^a^	9	52	5	6	0	11

Abbreviation: e-cigarettes, electronic cigarettes.

^a^ Other promotional items include but are not limited to t-shirts, hats, keychains, posters, and other items with tobacco companies’ insignia and logos.

Table 3 presents the results of our analysis showing the relationship between ownership of promotional material with tobacco initiation. In the unadjusted models (model 1), adolescents reporting ownership of ATP promotional materials were more than twice as likely to have initiated ATP use 1 year later (OR, 2.23; 95% CI, 1.26-3.97). Adjusting for individual demographic factors (model not shown) including age, sex, and race/ethnicity, adolescents owning ATP promotional items at baseline were more likely to initiate ATP use 1 year later compared with individuals not owning ATP promotional material (OR, 2.31; 95% CI, 1.28-4.18). After adjustment for all covariates (model 2), including age, sex, race/ethnicity, mothers’ educational level, baseline alcohol use ever, and baseline cigarette use ever, the association between ownership of ATP promotional material and ATP initiation in wave 2 yielded similar results, with greater odds of ATP initiation in wave 2 among individuals owning ATP promotional material compared with individuals not owning ATP promotional material (OR, 2.13; 95% CI, 1.16-3.91). In unadjusted models, ATP or cigarette initiation was significantly associated with ownership of ATP or cigarette promotional material. When covariates were considered, however, results were attenuated and not statistically significant. Specifically, results of adjusted models assessing the combined association of owning ATP or cigarette promotions with the combined outcome variable of ATP or cigarette initiation were not statistically significant (OR, 1.62; 95% CI, 0.91-2.91).

**Table 3.** Generalized Estimating Equation Logistic Regression Models of Ownership of ATP-Specific Promotional Materials (Wave 1) With Subsequent ATP Initiation (Wave 2) and Any Tobacco Initiation Among 757 Adolescents and Young Adults

Type of Promotional Material	OR (95% CI)			
	Model 1^a^		Model 2^b^	
	ATP Initiation^c^	ATP or Cigarette Initiation^d^	ATP Initiation	ATP or Cigarette Initiation
Ownership of ATP promotional material	2.23 (1.26-3.97)	1.51 (0.83-2.75)	2.13 (1.16-3.91)	1.42 (0.75-2.71)
Ownership of ATP or cigarette product promotional material	2.13 (1.26-3.60)	1.74 (1.02-2.96)	1.99 (1.11-3.56)	1.62 (0.91-2.91)

Abbreviations: ATP, alternative tobacco product; OR, odds ratio.

^a^ Model 1 is unadjusted for any covariate.

^b^ Model 2 presents a model adjusting for baseline age, sex, race/ethnicity, mother’s educational level, ever alcohol use at baseline, and ever cigarette use at baseline.

^c^ Wave 2 ATP initiation analysis sample size was 129.

^d^ Wave 2 ATP and cigarette initiation sample size was 141.

## Discussion

To our knowledge, this is the first study to examine the association of marketing receptivity, as defined by ownership of ATP promotional items, with subsequent initiation of ATP use including e-cigarettes; smokeless tobacco; tobacco pipes; cigars, cigarillos, or little cigars; and hookah among adolescents and young adults. In this study, among a sample of California adolescents and young adults, self-reported ownership of ATP promotional material and self-reported ownership of ATP- or cigarette-specific promotional material at baseline were longitudinally associated with ATP initiation 1 year later.

These findings are consistent with and extend the literature showing ownership of cigarette-related promotional material is associated with cigarette use among adolescents and young adults.^15,19,20^ In an analysis of 24 658 middle school and high school participants from the 2012 National Youth Tobacco Survey, exposure to tobacco coupons was found to be associated with higher likelihood of intending to purchase cigarettes in the next 30 days, revealing potential relationships of promotional materials and adolescent cigarette use.^19^ Moreover, exposure to these marketing strategies is associated with increased rates of cigarette use among nonsmokers, translating to faster escalation of cigarette use and lower smoking reduction.^20^ Similarly, our findings suggest that ATP-specific promotional materials may be associated with ATP use among adolescents and young adults; this is likely due to tobacco companies using strategies to market ATPs similar to those used to market traditional cigarettes.

This study’s findings are important given previous research showing that marketing receptivity is associated with ATP use^12,16^ and ATP use is associated with subsequent cigarette initiation.^24,35,36,37,38^ Use of ATPs is strongly associated with subsequent cigarette initiation and is also associated with higher odds of becoming an established smoker, even for adolescents and young adults who have an otherwise low risk for cigarette smoking.^35^ This shift to cigarette use after ATP use may be due to altered perceptions of cigarettes through increased ATP advertisement and marketing exposure.^39,40^ For example, exposure to marketing for ATPs such as e-cigarettes has been found to be associated with subsequent cigarette smoking, even though the promoted products are not cigarettes.^18^

Our study’s findings are also important given the restrictions currently implemented for tobacco advertising. Adolescents and young adults in our study are reporting marketing receptivity not only to unregulated ATPs but also to cigarettes. Despite restrictions on the distribution of promotional materials for cigarettes, adolescents and young adults still report owning promotional items for cigarettes. Thus, we believe the significant findings of ATP marketing receptivity positively associated with ATP initiation found in this study have important public health and regulatory implications. Increased ATP receptivity may not only increase the uptake of ATPs in adolescents, but it may simultaneously contribute to the renormalization of smoking. These results suggest the importance of regulating the distribution of all types of tobacco promotional materials to adolescents and young adults.

### Limitations

Our results should be interpreted in light of a few limitations. First, our study does not account for selection or attrition bias whereby adolescents and young adults who were differentially at higher risk for ATP initiation may have been more likely to participate in this study or be lost to follow-up. In addition, although we measured and adjusted for all the confounders we identified based on the literature, the potential influence of unmeasured confounders is another limitation. This study was underpowered to test effect modification by age and other covariates. Future studies should aim to assess these potential interactions. Moreover, no causal relationships can be established between ownership of promotional material and ATP use, although previous research has made a strong case for causal effect of tobacco marketing and initiation of cigarette use among adolescents and young adults.^2,26^ In addition, past 30-day ATP use was defined as any ATP use in the past 30 days, but because many of these ATPs are relatively new products, this measure may only be reflecting recent use of these products and not established use. Moreover, all measures in this study were self-reported, and our study does not account for information bias including potential measurement error. It is also possible that other mechanisms, including friends’ ATP use and individual alcohol use, may explain ATP initiation above and beyond ownership of promotional materials. Although we adjusted for baseline alcohol use in our analysis and yielded no significant results, future studies should further examine these relationships. Furthermore, our results may not be generalizable to adolescents and young adults throughout and outside of California. However, rates of tobacco and ATP use in our study are consistent with national rates for youth.

## Conclusions

Our results are consistent with the suggestion that regulating the distribution of promotional materials for ATP products would likely result in a significant reduction in ATP use. This study fills gaps in the literature by simultaneously assessing a wide variety of ATPs used by adolescents and young adults and extending analyses of the association between marketing receptivity and tobacco initiation to include ownership of promotional materials. Identifying factors associated with ATP use can help inform tobacco control campaigns and ATP regulations such as restrictions for materials distributed to adolescents. The results of this study will hopefully inform the US Food and Drug Administration’s approach to regulating ATPs and their corresponding marketing efforts. Current restrictions of cigarette samples, coupons, and other promotional material distributed to adolescents and young adults should extend to ATPs.

## References

[1] Johnston LD, Miech RA, O’Malley PM, Bachman JG, Schulenberg JE, Patrick ME Monitoring the Future: National Survey Results on Drug Use 2017 Overview, Key Findings on Adolescent Drug Use. Ann Arbor, MI: Institute for Social Research; 2018. doi:10.3998/2027.42/148123

[2] US Department of Health and Human Services The Health Consequences of Smoking—50 Years of Progress: A Report of the Surgeon General. Atlanta, GA: US Department of Health and Human Services, Centers for Disease Control and Prevention, National Center for Chronic Disease Prevention and Health Promotion, Office on Smoking and Health; 2014.

[3] Wang TW, Gentzke A, Sharapova S, Cullen KA, Ambrose BK, Jamal A Tobacco product use among middle and high school students—United States, 2011-2017. MMWR Morb Mortal Wkly Rep. 2018;67(22):-. doi:10.15585/mmwr.mm6722a3 29879097 PMC5991815

[4] Cullen KA, Ambrose BK, Gentzke AS, Apelberg BJ, Jamal A, King BA Notes from the field: use of electronic cigarettes and any tobacco product among middle and high school students—United States, 2011-2018. MMWR Morb Mortal Wkly Rep. 2018;67(45):1276-1277. doi:10.15585/mmwr.mm6745a5 30439875 PMC6290807

[5] Wang TW, Asman K, Gentzke AS, Tobacco product use among adults—United States, 2017. MMWR Morb Mortal Wkly Rep. 2018;67(44):1225-1232. doi:10.15585/mmwr.mm6744a2 30408019 PMC6223953

[6] Roditis M, Delucchi K, Cash D, Halpern-Felsher B Adolescents’ perceptions of health risks, social risks, and benefits differ across tobacco products. J Adolesc Health. 2016;58(5):558-566. doi:10.1016/j.jadohealth.2016.01.012 27107909 PMC5072979

[7] Fairchild AL, Bayer R, Colgrove J The renormalization of smoking? e-cigarettes and the tobacco endgame. N Engl J Med. 2014;370(4):293-295. doi:10.1056/NEJMp1313940 24350902

[8] Gorukanti A, Delucchi K, Ling P, Fisher-Travis R, Halpern-Felsher B Adolescents’ attitudes towards e-cigarette ingredients, safety, addictive properties, social norms, and regulation. Prev Med. 2017;94:65-71. doi:10.1016/j.ypmed.2016.10.019 27773711 PMC5373091

[9] Singh T, Arrazola RA, Corey CG, Tobacco use among middle and high school students—United States, 2011-2015. MMWR Morb Mortal Wkly Rep. 2016;65(14):361-367. doi:10.15585/mmwr.mm6514a1 27077789

[10] Richardson A, Ganz O, Vallone D Tobacco on the web: surveillance and characterisation of online tobacco and e-cigarette advertising. Tob Control. 2015;24(4):341-347. doi:10.1136/tobaccocontrol-2013-051246 24532710

[11] Duke JC, Lee YO, Kim AE, Exposure to electronic cigarette television advertisements among youth and young adults. Pediatrics. 2014;134(1):e29-e36. doi:10.1542/peds.2014-0269 24918224

[12] Thrul J, Lisha NE, Ling PM Tobacco marketing receptivity and other tobacco product use among young adult bar patrons. J Adolesc Health. 2016;59(6):642-647. doi:10.1016/j.jadohealth.2016.08.008 27707516 PMC5123918

[13] Jackler RK, Li VY, Cardiff RAL, Ramamurthi D Promotion of tobacco products on Facebook: policy versus practice. Tob Control. 2019;28(1):67-73. 29622602 10.1136/tobaccocontrol-2017-054175

[14] Mackey TK, Miner A, Cuomo RE Exploring the e-cigarette e-commerce marketplace: identifying internet e-cigarette marketing characteristics and regulatory gaps. Drug Alcohol Depend. 2015;156:97-103. doi:10.1016/j.drugalcdep.2015.08.032 26431794

[15] Tessman GK, Caraballo RS, Corey CG, Xu X, Chang CM Exposure to tobacco coupons among U.S. middle and high school students. Am J Prev Med. 2014;47(2)(suppl 1):S61-S68. doi:10.1016/j.amepre.2014.05.001 25044197 PMC4624109

[16] Pierce JP, Sargent JD, Portnoy DB, Association between receptivity to tobacco advertising and progression to tobacco use in youth and young adults in the PATH study. JAMA Pediatr. 2018;172(5):444-451. doi:10.1001/jamapediatrics.2017.5756 29582078 PMC5875336

[17] Pierce JP, Sargent JD, White MM, Receptivity to tobacco advertising and susceptibility to tobacco products. Pediatrics. 2017;139(6):e20163353. doi:10.1542/peds.2016-3353 28562266 PMC5470502

[18] Cruz TB, McConnell R, Low BW, Tobacco marketing and subsequent use of cigarettes, e-cigarettes and hookah in adolescents [published online May 28, 2018]. Nicotine Tob Res. doi:10.1093/ntr/nty107 29846704 PMC6588392

[19] Choi K The associations between exposure to tobacco coupons and predictors of smoking behaviours among US youth. Tob Control. 2016;25(2):232-235. doi:10.1136/tobaccocontrol-2014-052147 25882686 PMC4608847

[20] Choi K, Forster J Tobacco direct mail marketing and smoking behaviors in a cohort of adolescents and young adults from the U.S. upper Midwest: a prospective analysis. Nicotine Tob Res. 2014;16(6):886-889. doi:10.1093/ntr/ntu013 24532353 PMC4015100

[21] Soneji S, Ambrose BK, Lee W, Sargent J, Tanski S Direct-to-consumer tobacco marketing and its association with tobacco use among adolescents and young adults. J Adolesc Health. 2014;55(2):209-215. doi:10.1016/j.jadohealth.2014.01.019 24661738 PMC4241586

[22] MacFadyen L, Hastings G, MacKintosh AM Cross-sectional study of young people’s awareness of and involvement with tobacco marketing. BMJ. 2001;322(7285):513-517. doi:10.1136/bmj.322.7285.513 11230063 PMC26553

[23] Williams RS VapeCons: e-cigarette user conventions. J Public Health Policy. 2015;36(4):440-451. doi:10.1057/jphp.2015.31 26424201 PMC4641520

[24] Richardson A, Williams V, Rath J, Villanti AC, Vallone D The next generation of users: prevalence and longitudinal patterns of tobacco use among US young adults. Am J Public Health. 2014;104(8):1429-1436. doi:10.2105/AJPH.2013.301802 24922152 PMC4103202

[25] Healton C The tobacco Master Settlement Agreement—strategic lessons for addressing public health problems. N Engl J Med. 2018;379(11):997-1000. doi:10.1056/NEJMp1802633 30207911

[26] DiFranza JR, Wellman RJ, Sargent JD, Weitzman M, Hipple BJ, Winickoff JP; Tobacco Consortium, Center for Child Health Research of the American Academy of Pediatrics Tobacco promotion and the initiation of tobacco use: assessing the evidence for causality. Pediatrics. 2006;117(6):e1237-e1248. doi:10.1542/peds.2005-1817 16740823

[27] Food and Drug Administration; Department of Health and Human Services Deeming tobacco products to be subject to the Federal Food, Drug, and Cosmetic Act, as amended by the Family Smoking Prevention and Tobacco Control Act; restrictions on the sale and distribution of tobacco products and required warning statements for tobacco products; final rule. Fed Regist. 2016;81(90):28973-29106. 27192730

[28] Vandenbroucke JP, von Elm E, Altman DG, ; STROBE Initiative Strengthening the Reporting of Observational Studies in Epidemiology (STROBE): explanation and elaboration. Int J Surg. 2014;12(12):1500-1524. doi:10.1016/j.ijsu.2014.07.014 25046751

[29] Roditis ML, Delucchi K, Chang A, Halpern-Felsher B Perceptions of social norms and exposure to pro-marijuana messages are associated with adolescent marijuana use. Prev Med. 2016;93:171-176. doi:10.1016/j.ypmed.2016.10.013 27746339 PMC5268762

[30] Ed Data Education Data Partnership website https://www.ed-data.org/article/The-Ed-Data-Partnership. Accessed March 5, 2018.

[31] McKelvey K, Halpern-Felsher B Adolescent cigarette smoking perceptions and behavior: tobacco control gains and gaps amidst the rapidly expanding tobacco products market from 2001 to 2015. J Adolesc Health. 2017;60(2):226-228. doi:10.1016/j.jadohealth.2016.09.025 27939880 PMC5270372

[32] Zeger SL, Liang KY, Albert PS Models for longitudinal data: a generalized estimating equation approach. Biometrics. 1988;44(4):1049-1060. doi:10.2307/2531734 3233245

[33] Hubbard AE, Ahern J, Fleischer NL, To GEE or not to GEE: comparing population average and mixed models for estimating the associations between neighborhood risk factors and health. Epidemiology. 2010;21(4):467-474. doi:10.1097/EDE.0b013e3181caeb90 20220526

[34] Hanley JA, Negassa A, Edwardes MD, Forrester JE Statistical analysis of correlated data using generalized estimating equations: an orientation. Am J Epidemiol. 2003;157(4):364-375. doi:10.1093/aje/kwf215 12578807

[35] Chaffee BW, Watkins SL, Glantz SA Electronic cigarette use and progression from experimentation to established smoking. Pediatrics. 2018;141(4):e20173594. doi:10.1542/peds.2017-3594 29507167 PMC5869336

[36] Rath JM, Villanti AC, Abrams DB, Vallone DM Patterns of tobacco use and dual use in US young adults: the missing link between youth prevention and adult cessation. J Environ Public Health. 2012;2012:679134. doi:10.1155/2012/679134 22666279 PMC3361253

[37] Wang B, King BA, Corey CG, Arrazola RA, Johnson SE Awareness and use of non-conventional tobacco products among U.S. students, 2012. Am J Prev Med. 2014;47(2)(suppl 1):S36-S52. doi:10.1016/j.amepre.2014.05.003 25044194 PMC4519346

[38] Dutra LM, Glantz SA Electronic cigarettes and conventional cigarette use among US adolescents: a cross-sectional study. JAMA Pediatr. 2014;168(7):610-617. doi:10.1001/jamapediatrics.2013.5488 24604023 PMC4142115

[39] Gibson LA, Creamer MR, Breland AB, Measuring perceptions related to e-cigarettes: important principles and next steps to enhance study validity. Addict Behav. 2018;79:219-225. doi:10.1016/j.addbeh.2017.11.017 29175027 PMC5807230

[40] Kim M, Popova L, Halpern-Felsher B, Ling PM Effects of e-cigarette advertisements on adolescents’ perceptions of cigarettes. Health Commun. 2019;34(3):290-297. doi:10.1080/10410236.2017.1407230 29236550 PMC5999542

